# Ensuring safety and feasibility for resection of pediatric benign ovarian tumors by single-port robot-assisted laparoscopic surgery using the da Vinci Xi system

**DOI:** 10.3389/fsurg.2022.944662

**Published:** 2022-08-19

**Authors:** Deqiang Xu, Heyun Gao, Shanzhen Yu, Guangbin Huang, Dan Lu, Kun Yang, Wei Zhang, Wen Zhang

**Affiliations:** ^1^ Department of Pediatric Surgery, Zhongnan Hospital of Wuhan University, Wuhan, China; ^2^ Department of Ultrasound Imaging, Zhongnan Hospital of Wuhan University, Wuhan, China; ^3^ Department of Urology, Zhongnan Hospital of Wuhan University, Wuhan, China; ^4^ Department of Gynaecology, Zhongnan Hospital of Wuhan University, Wuhan, China

**Keywords:** single-port robot-assisted laparoscopic surgery, pediatric, resection, benign ovarian tumors, da Vinci Xi

## Abstract

**Background:**

Single-port robot-assisted laparoscopic surgery (S-RALS) is rarely applied in pediatric surgery. There is still no study on the application of S-RALS for resection of pediatric benign ovarian tumors. The current study aimed to investigate the safety and feasibility of S-RALS for resection of pediatric benign ovarian tumors using the da Vinci Xi system.

**Methods:**

The clinical data of three patients who underwent S-RALS for resection of benign ovarian tumors in the Department of Pediatric Surgery, Zhongnan Hospital of Wuhan University from May 2020 to September 2021 were retrospectively analyzed. The mean age of these children was 7.9 years (5.8–9.3 years). One was a case of bilateral ovarian tumors, and the other two were cases of right ovarian tumors.

**Results:**

All three patients successfully underwent the resection of ovarian tumors through S-RALS without conversion to laparotomy. The average operation time was 180 min (118–231 min). The average amount of blood loss was 20 ml (10–35 ml). No drainage tube was placed. All postoperative pathological types of ovarian tumors were mature cystic teratomas in the three cases. All patients started a liquid diet 2 h after surgery. The average length of postoperative hospital stay was 4.7 days (3–7 days). No tumor recurred, no surgical site hernia occurred, and the wound healed very well with a cosmetic scar in the lower umbilical crease during the postoperative follow-up for 6–18 months.

**Conclusion:**

S-RALS has the advantages of less surgical trauma, quick postoperative recovery, and a cosmetic scar in the lower umbilical crease. It is safe, effective, and feasible for pediatric benign ovarian tumors.

## Introduction

Ovarian tumors are common pediatric female reproductive system tumors, and most are mature cystic teratomas, which belong to benign tumors (1). The preferred treatment for benign ovarian tumors is ovarian sparing tumorectomy (2). At present, laparoscopic technology has been widely used in pediatric operations because of its good cosmetic effect, small intraoperative trauma, and quick postoperative recovery (3, 4). However, it still has limitations such as increased tremors, a long learning curve, and operator fatigue when a long time of surgery (5). In recent years, with the rapid development of minimally invasive surgery, robotic surgery systems have been gradually used in many children's surgical operations (6). Single-port laparoscopic surgery (SPLS) has a better cosmetic effect because it reduces the incision on the abdominal surface through the natural cavity of the human body. However, single-port robot-assisted laparoscopic surgery (S-RALS) is rarely applied in pediatric surgery due to the small abdominal space and thin abdominal wall in children. Until today, only a few studies reported the applications of S-RALS in children, such as pyeloplasty (7), cholecystectomy (8), and splenectomy (9). There is no study on the application of S-RALS for resection of pediatric benign ovarian tumors. This study retrospectively analyzed the clinical data of three patients who underwent S-RALS for resection of benign ovarian tumors in the Department of Pediatric Surgery, Zhongnan Hospital of Wuhan University, to investigate the feasibility and safety of this technology in children with benign ovarian tumors. Meanwhile, we provided the preliminary experience of S-RALS for resection of pediatric benign ovarian tumors.

## Materials and methods

### Clinical data

**Figure 1:**
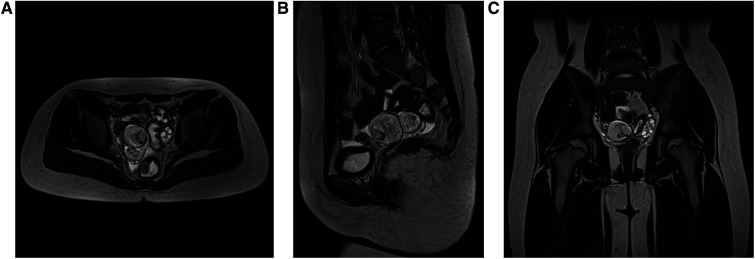
Representative preoperative MRI of patients: (**A**) horizontal plane, (**B**) sagittal plane, and (**C**) coronal plane. MRI indicated that the ovarian tumor was benign. MRI, magnetic resonance imaging.

The clinical data of three patients who underwent S-RALS for resection of benign ovarian tumors in the Department of Pediatric Surgery, Zhongnan Hospital of Wuhan University from May 2020 to September 2021 were retrospectively analyzed. All preoperative ultrasound imaging, computerized tomography (CT), or magnetic resonance imaging (MRI) results (Figure 1) and tumor markers (Table 1) indicated these ovarian tumors were benign. All clinical data were obtained after the approval of the Hospital Committee for Investigation in Humans (No. 2022003K). Written informed consent to participate in this study was provided by the participants’ legal guardians.

**Table 1:** Preoperative tumor markers of patients.

Patient	AFP (ng/ml)	CA125 (U/ml)	β-HCG (mIU/ml)	NSE (µg/l)	VMA
1	1.38	28.47	1.20	19.90	Negative
2	0.89	11.39	<0.30	19.30	Negative
3	1.11	12.97	0.78	13.59	Negative

AFP, alpha fetoprotein; NSE, neuron-specific enolase; VMA, vanillylmandelic acid

### Surgical methods

**Figure 2:**
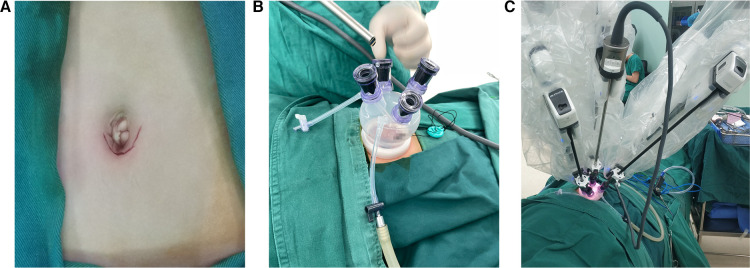
Appearance of a single incision and the placement of robotic instruments: (**A**) appearance of the single incision, (**B**) special laparoscopic device with a single port and four channels, and (**C**) placement of the 30° lens and two manipulators, forming a stable triangle relationship.

**Figure 3:**
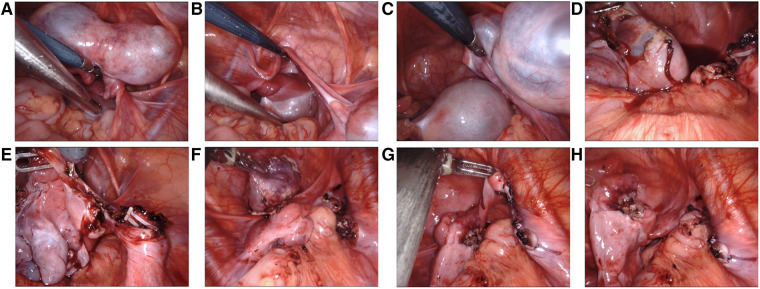
Surgical procedures of S-RALS for resection of ovarian tumors: (**A,B**) exposing the diseased ovaries fully. The images indicate bilateral ovarian torsion, (**C**) resetting the ovarian torsion, (**D**) opening the ovaries and exposing the tumor tissues, (**E,F**) removing the tumor along the tumor margin completely, and (**G,H**) suturing the ovarian tissues by 5-0 absorbable suture line. S-RALS, single-port robot-assisted laparoscopic surgery.

(i) Body position: After general anesthesia and endotracheal intubation, the child was placed in supine and Trendelenburg position with an indwelling catheter.
(ii) Placement of the mechanical arm: After conventional disinfection, a 2.5 cm arc-shaped incision was made through the lower edge of the umbilical ring (Figure 2A) and then a special laparoscopic device, which consists of a single port and four channels, was inserted (Figure 2B). CO_2_ artificial pneumoperitoneum was established with a pressure of 6–8 mmHg (1 mmHg = 0.133 kPa). A 30° lens and two manipulators were placed into the laparoscopic device, respectively, and the two manipulators formed a stable triangular relationship with the lens (Figure 2C).
(iii) Surgical procedures: Moving away the bowels surrounding the affected ovary, exposing the diseased ovaries fully (Figures 3A,B), resetting the ovarian torsion if occurred (Figure 3C), opening the ovaries and exposing the tumor tissues (Figure 3D), removing the tumors along the tumor margin completely (Figures 3E,F), hemostasis for the wound, suturing the ovarian tissues by the 5-0 absorbable suture line (Figures 3G,H), picking up the tumor tissues into the specimen bag, removing the robotic device, taking out tumor tissues through the umbilical incision, making postoperative pathological examination, washing the abdominal cavity with distilled water, closing the abdomen layer by layer, and restoring the normal structure of the umbilicus.

## Results

**Figure 4:**
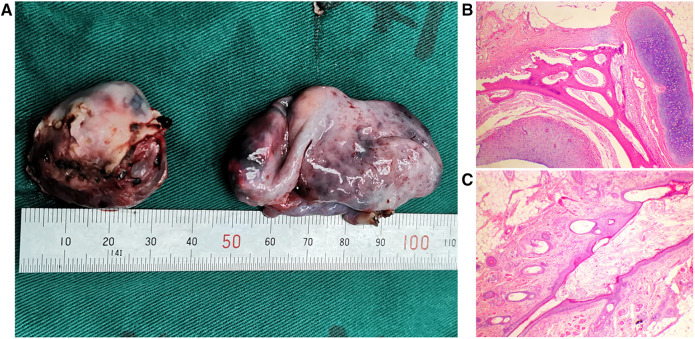
Typical photograph and pathological examination of ovarian tumors: (**A**) typical photograph of ovarian tumors and (**B,C**) pathological examination of ovarian tumors. The images indicated mature cystic teratomas.

**Figure 5:**
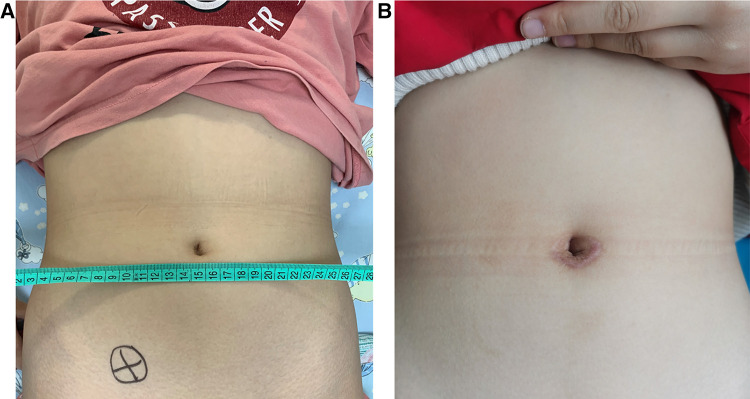
Appearance of umbilicus: (**A**) the appearance of preoperative umbilicus and (**B**) the appearance of postoperative umbilicus after one year.

All three patients successfully underwent the resection of ovarian tumors through S-RALS without conversion to laparotomy. As shown in Table 2, the average operation time was 146 min (118–181 min), the average amount of blood loss was 20 ml (10–35 ml), and no drainage tube was placed. All postoperative pathological types of ovarian tumors were mature cystic teratomas in the three cases (Figure 4; Table 2). All patients started a liquid diet 2 h after surgery. The average length of postoperative hospital stay was 4.7 days (3–7 days). No tumor recurred, no surgical site hernia occurred, and the wound healed very well with a cosmetic scar in the lower umbilical crease (Figure 5) during the postoperative follow-up for 6–18 months.

**Table 2:** Data of patients.

Patient	Side	Age (years)	Hemorrhage (ml)	Operating time (min)	Drainage	Postoperative pathological type	Postoperative hospital stay (days)	Follow-up (months)
1	Bilateral	5.8	35	181	No	Mature cystic teratoma	7	18
2	Right	8.6	10	118	No	Mature cystic teratoma	4	12
3	Right	9.3	15	140	No	Mature cystic teratoma	3	3

## Discussion

Benign mature ovarian teratoma is the most common ovarian tumor in children, which usually requires operative management (10, 11). However, unilateral oophorectomy at a young age may result in a shorter reproductive life span and early menopause (12). To spare future fertility, it has been suggested that ovarian sparing tumorectomy should be the first-line procedure in children for the treatment of benign ovarian tumors (2). Laparoscopic surgery is considered the gold standard for managing benign ovarian tumors (13). The use of the laparoscopic technique was associated with the higher preservation of ovarian tissue (11). Meanwhile, studies suggested that laparoscopic surgery for pediatric benign ovarian tumors has the advantages of shorter length of stay, decreased postoperative pain, decreased recovery time, and less adhesion formation compared with open surgical techniques (14, 15).

However, laparoscopic surgery may have difficulty peeling off the cyst wall completely because of poor visual field stability and amplification of tremors (16). At the same time, it needs a long learning curve and the surgeons are easy to be fatigued (17). Robot-assisted laparoscopic surgery (RALS) can solve the problem due to stable and accurate operation and highly magnified three-dimensional vision (18, 19). Compared with laparoscopic surgery, it needs a shorter learning curve and the surgeons are no longer fatigued (20, 21). Until now, RALS has been gradually applied to pediatric reconstructive surgery, such as pyeloplasty (22), ureterovesical replantation (23), resection of ovarian tumors (24), and so on.

As the extension of laparoscopic surgery, transumbilical SPLS can improve the cosmesis by reducing the number of trocar incisions (25). However, it has a limitation of the constraint of operating triangle at the time of operation (25). S-RALS, as a combination of RALS and SPLS, recovers the structure of the operating triangle and saves the cosmetic advantage of SPLS (26). It has been applied in adult surgery including gynecology and urology in recent years. However, only a few studies reported the application of S-RALS in pediatric surgery since the small abdominal space limits its application. For example, Mattei (27) reported 20 cases of single-site robot-assisted laparoscopic cholecystectomy in children and adolescents in 2017. Kang et al. (28) reported a case of robot-assisted laparoscopic single-port pyeloplasty using the da Vinci SP system in 2019. There have been no studies on S-RALS in pediatric ovarian sparing tumorectomy until now, especially using da Vinci Xi.

In this study, we report S-RALS in the resection of pediatric benign ovarian tumors using da Vinci Xi for the first time. Ovarian tumors were found in three patients with symptoms of abdominal pain; therefore, we chose surgery for them instead of observation. According to the previous study, the tumor is considered benign when it is cystic, smaller than 10 cm, and not associated with positive tumor markers; otherwise, it is perhaps malignant (11). We performed CT or MRI before surgery; all images of the three patients indicated that the tumors were cystic and smaller than 10 cm. Meanwhile, all tumor markers were negative. Based on these preoperative examinations, we performed S-RALS for resection of benign ovarian tumors using da Vinci Xi. No conversion to laparotomy occurred in our study because no malignant signs were found intraoperatively. A successful operation with S-RALS requires rich experience in SPLS and RALS for surgeons. We have operated numbers of SPLS and completed about 100 cases of RALS including 20 cases of S-RALS until now; the experience of SPLS has been reported in our previous studies (29, 30). We made a single incision of 2.5 cm through the lower edge of the umbilical ring and placed a special single port with four channels in all three cases. The incision length is similar or even shorter compared with the method of making three or four 5 mm ports; moreover, it is more cosmetic by hiding the wound under the umbilicus. Despite the thin abdominal wall in children, no postoperative surgical site hernia occurred, as we did not cut the muscle when we made the incision through the lower edge of the umbilical ring. It is consistent with the advantages of SPLS for the resection of pediatric benign ovarian tumors (31, 32). Moreover, we found that it is easier to resect pediatric benign ovarian tumors through S-RALS compared with SPLS because of the recovery of the triangulation. The key point is to cut the ovary using scissors without electricity when exposing and dissecting the tumor, which could prevent the ovary from heat injury. The use of the laparoscopic technique was associated with higher preservation of ovarian tissue (11); we also preserved the normal ovarian tissue as more as possible. Although ovarian cystectomy does not need to suture the ovarian tissue, we sutured the ovarian tissue and closed the cavity to preserve the ovarian morphology and reduce recurrence. Moreover, we aspirated the cyst fluid with a puncture outfit if the tumor was too large, protecting it from the spillage of the cyst fluid. Some reports demonstrated that spillage of the cyst fluid occurred in laparoscopic cases when dissecting the tumor (2, 33); we have no case of the spillage occurring in the study. The main reason is that the da Vinci Xi robotic system is very stable apart from our operation of aspirating the cyst fluid, but more cases need to be investigated. In our study, one case had more hemorrhage and operative time compared to the other two; this is because the case consisted of bilateral tumors. To enhance the recovery after surgery, all of the patients started a liquid diet 2 h after surgery and no abdominal drainage tube was placed. The average length of postoperative hospital stay was 4.7 days (3–7 days), which is associated with the lack of primary medical resources and COVID-2019 in our country.

In summary, this is the first study to report S-RALS in the resection of pediatric benign ovarian tumors. S-RALS has the advantages of less surgical trauma, quick postoperative recovery, and a cosmetic scar in the lower umbilical crease. It is safe, effective, and feasible for pediatric benign ovarian tumors. However, further good quality randomized controlled trials are needed to suggest S-RALS as a suitable approach for pediatric benign ovarian tumors.

## Data Availability

The original contributions presented in the study are included in the article/Supplementary Material, further inquiries can be directed to the corresponding author.
